# Overabundance of *Asaia* and *Serratia* Bacteria Is Associated with Deltamethrin Insecticide Susceptibility in *Anopheles coluzzii* from Agboville, Côte d’Ivoire

**DOI:** 10.1128/Spectrum.00157-21

**Published:** 2021-10-20

**Authors:** Bethanie Pelloquin, Mojca Kristan, Constant Edi, Anne Meiwald, Emma Clark, Claire L. Jeffries, Thomas Walker, Nsa Dada, Louisa A. Messenger

**Affiliations:** a Department of Disease Control, Faculty of Infectious and Tropical Diseases, London School of Hygiene and Tropical Medicine, London, United Kingdom; b School of Tropical Medicine and Global Health, University of Nagasaki, Nagasaki, Japan; c Centre Suisse de Recherche Scientifique en Côte d’Ivoire, Abidjan, Côte d’Ivoire; d Faculty of Science and Technology, Norwegian University of Life Sciences, Aas, Norway; e Public Health and Epidemiology Department, Nigerian Institute of Medical Research, Lagos, Nigeria; f Tropical Infectious Disease Research Center, University of Abomey-Calavi, Cotonou, Benin; Georgia Institute of Technology

**Keywords:** *Anopheles coluzzii*, insecticide resistance, microbiota, deltamethrin, malaria, Côte d’Ivoire, *Asaia*, *Serratia*

## Abstract

Insecticide resistance among mosquito species is now a pervasive phenomenon that threatens to jeopardize global malaria vector control efforts. Evidence of links between the mosquito microbiota and insecticide resistance is emerging, with significant enrichment of insecticide degrading bacteria and enzymes in resistant populations. Using 16S rRNA amplicon sequencing, we characterized and compared the microbiota of Anopheles coluzzii in relation to their deltamethrin resistance and exposure profiles. Comparisons between 2- and 3-day-old deltamethrin-resistant and -susceptible mosquitoes demonstrated significant differences in microbiota diversity. *Ochrobactrum*, *Lysinibacillus*, and *Stenotrophomonas* genera, each of which comprised insecticide-degrading species, were significantly enriched in resistant mosquitoes. Susceptible mosquitoes had a significant reduction in alpha diversity compared to resistant individuals, with *Asaia* and *Serratia* dominating microbial profiles. There was no significant difference in deltamethrin-exposed and -unexposed 5- to 6-day-old individuals, suggesting that insecticide exposure had minimal impact on microbial composition. *Serratia* and *Asaia* were also dominant in 5- to 6-day-old mosquitoes, which had reduced microbial diversity compared to 2- to 3-day-old mosquitoes. Our findings revealed significant alterations of Anopheles coluzzii microbiota associated with deltamethrin resistance, highlighting the potential for identification of novel microbial markers for insecticide resistance surveillance. qPCR detection of *Serratia* and *Asaia* was consistent with 16S rRNA sequencing, suggesting that population-level field screening of bacterial microbiota may be feasibly integrated into wider resistance monitoring, if reliable and reproducible markers associated with phenotype can be identified.

**IMPORTANCE** Control of insecticide-resistant vector populations remains a significant challenge to global malaria control and while substantial progress has been made elucidating key target site mutations, overexpressed detoxification enzymes and alternate gene families, the contribution of the mosquito microbiota to phenotypic insecticide resistance has been largely overlooked. We focused on determining the effects of deltamethrin resistance intensity on Anopheles coluzzii microbiota and identifying any microbial taxa associated with phenotype. We demonstrated a significant reduction in microbial diversity between deltamethrin-resistant and -susceptible mosquitoes. Insecticide degrading bacterial species belonging to *Ochrobactrum*, *Lysinibacillus*, and *Stenotrophomonas* genera were significantly enriched in resistant mosquitoes, while *Asaia* and *Serratia* dominated microbial profiles of susceptible individuals. Our results revealed significant alterations of Anopheles coluzzii microbiota associated with deltamethrin resistance, highlighting the potential for identification of novel microbial markers for surveillance and opportunities for designing innovative control techniques to prevent the further evolution and spread of insecticide resistance.

## INTRODUCTION

Malaria remains a considerable public health problem with an estimated 229 million cases worldwide, including 409,000 deaths in 2019 alone (1). Malaria mortality has fallen since 2010, largely due to the scale-up of treatment, diagnostics, and insecticide-based vector control interventions, principally long-lasting insecticidal nets (LLINs) and indoor residual spraying. However, global gains in malaria control have begun to stall (2). Insecticide resistance among major malaria vector species is now a pervasive phenomenon, affecting more than 90% of countries with ongoing transmission (2). Of particular concern is the continued spread of resistance to pyrethroids, which were until recently, the only class of insecticide recommended for use in LLINs. Pyrethroids are still a crucial component of next-generation LLINs (3), and resistance may severely threaten the long-term effectiveness of contemporary vector control programs.

Control of insecticide-resistant vector populations is predicated on a clear understanding of the complex interplay between molecular mechanisms and fitness costs which contribute to mosquito behavior, phenotype and vectorial capacity, the genetic and local environmental factors driving ongoing resistance selection, and the implications of resistance for intervention operational efficacy. Substantial progress has been made elucidating key target site mutations (4–7), overexpression of detoxification enzymes (8–12) and alternate gene families and pathways (13–17), all of which play important roles in resistance modulation. Furthermore, the recent publication of genome data for more than 1,000 *Anopheles* (*An*.) *gambiae sensu lato* (s.l.) has illustrated the considerable genetic diversity among natural vector populations, raising concerns for the rapid evolution and spread of novel resistance mechanisms (18, 19).

In addition to host-mediated resistance mechanisms, evidence is emerging that changes in mosquito microbiota may confer resistance to certain insecticides. The mosquito microbiota is a heterogenous and variable network of microorganisms, comprising the bacterial, archaeal, viral, fungal, and other eukaryotic microbial communities which inhabit the mosquito cuticle and internal structures such as the midgut, salivary glands, and ovaries. Constituents of the microbiota can be either inherited from mother to offspring (20) or acquired from the environment, predominantly the larval habitat (21). Characterization of the microbiota in mosquitoes has shown varied phenotypic impacts on the host species, including on fitness (22), blood feeding (23), fecundity (24), immunity (25, 26), pathogen infection (27–33), and transmission (34). There is increasing interest in investigating symbionts of mosquito vectors because they may offer unique transmission-blocking opportunities. Similarly, studies on the role played by mosquito symbionts in insecticide resistance may offer a better understanding of the underlying mechanisms and the potential for designing innovative control techniques (35) and developing new insecticide resistance monitoring tools.

The interaction between insecticide resistance and arthropod microbiota has been examined principally in agricultural pest species. Chlorpyrifos (organophosphate)- and fipronil (phenylpyrazole)-resistant strains of the Diamondback moth, *Plutella xylostella* were shown to have a higher proportion of *Lactobacillales*, *Pseudomonadales*, and *Xanthomonadales* bacteria (36). Furthermore, the bean bug *Riptortus pedestris* and allied stinkbug species harbor symbiotic *Burkholderia* bacteria which degrade fenitrothion (organophosphate) and are present in greater abundance when this insecticide is applied to their habitat (37). As advanced molecular technologies become increasingly accessible, research in this area is expanding to disease vectors, with recent studies on several mosquito species. Whole-metagenome sequencing of microbiota from wild-caught fenitrothion-resistant and -susceptible *An. albimanus* mosquitoes showed distinct differences between these two groups (38). Fenitrothion-resistant mosquitoes had significant enrichment of organophosphate degrading bacteria and enzymes such as hydrolases, carboxylesterases, and phosphomonoesterases. Resistant mosquitoes also had lower bacterial diversity, with an overabundance of Klebsiella spp. and a reduction in the relative abundance of Enterobacter spp. It was suggested that selection for organophosphate-degrading bacteria may have developed alongside resistance, potentially in response to prior insecticide exposure (38). F_1_ progeny of field-caught *An. albimanus* exposed to the pyrethroids alpha-cypermethrin and permethrin had significantly greater abundance of bacteria from the genus Pseudomonas, of which several strains have been shown to metabolize pyrethroids, and from the genus *Pantoea* (39), which had previously been identified in insecticide-resistant mosquitoes (38). Pseudomonas, alongside *Clostridium* and *Rhizobium* species, were also implicated in lambda-cyhalothrin (pyrethroid) resistance in wild populations of *Aedes aegypti* (*Ae. aegypti*) from Colombia (40). Addition of tetracycline to temephos-resistant (organophosphate) strains of *An. stephensi* destroyed the bacterial component of the microbiota and significantly reduced the activity of three main resistance enzymes: α esterase, glutathione *S*-transferase, and acetylcholinesterase, restoring mosquito susceptibility (41). Similarly, sterilization of *An. arabiensis* gut microbiota by antibiotics resulted in a decreased tolerance to deltamethrin (pyrethroid) and malathion (organophosphate) (42).

To date, information on field populations of the *An. gambiae* complex, the main malaria vectors in sub-Saharan Africa, is limited to recent reports of significant enrichment of known pyrethroid degrading taxa (*Sphingobacterium*, *Lysinibacillus*, and Streptococcus) in permethrin-resistant *An. gambiae sensu stricto* (s.s.) from Kenya (43). To address this deficit, we comparatively characterized the bacterial microbiota of *An. coluzzii*, collected from an area of high pyrethroid resistance in Côte d’Ivoire. We specifically focused on determining the effects of deltamethrin resistance intensity on host microbiota and identifying any microbial taxa associated with resistance phenotypes.

## RESULTS

### Species identification and deltamethrin resistance profiles.

In total, 580 blood-fed *An. gambiae* s.l. were collected from Agboville using human landing catches (HLCs) during the rainy season in July 2019. Of these, 245 (42%) laid eggs via forced oviposition. Following larval development, 1,015 F_1_ *An. gambiae* s.l. pupae were identified as female and tested in deltamethrin resistance intensity assays as 2- to 3-day-old adults. Individuals were classified as susceptible if they were knocked down after exposure to 1× deltamethrin, resistant if they survived 60 min (2 to 3 days old) or 72 h (5 to 6 days old) postexposure to 1×, 5×, or 10× deltamethrin, or controls if they were unexposed to insecticide (comprising a mix of age-matched individuals of unknown phenotype). A total of 380 mosquitoes were randomly selected for DNA extraction, across all exposure and time groups, with 338 individuals identified as *An. coluzzii* (78.3%). From the remaining individuals, 31 were *An. gambiae* s.s. (8.1%), 10 failed to amplify (2.6%), and one individual was an *An. gambiae* s.s.–*An. coluzzii* hybrid (0.26%). Table S1 summarizes the number of mosquitoes selected for DNA extraction, pooling, and sequencing.

### Sequencing metrics.

A total of 1,156,076 reverse reads were obtained from sequencing. Quality control and denoising resulted in 2,999 unique amplicon sequence variants (ASVs), 878,155 in total. Filtering of ASVs associated with water and ethanol blanks, low-frequency ASVs, and ASVs not classified to the phylum level resulted in 210 unique ASVs, totaling 556,254 across 94 pools of mosquitoes. Table S3 summarizes the number of sequences processed per sample and the number of reads remaining after denoising and filtering.

### Susceptible *An. coluzzii* had microbiota which were significantly different to, and less diverse than, resistant mosquitoes.

**FIG 1:**
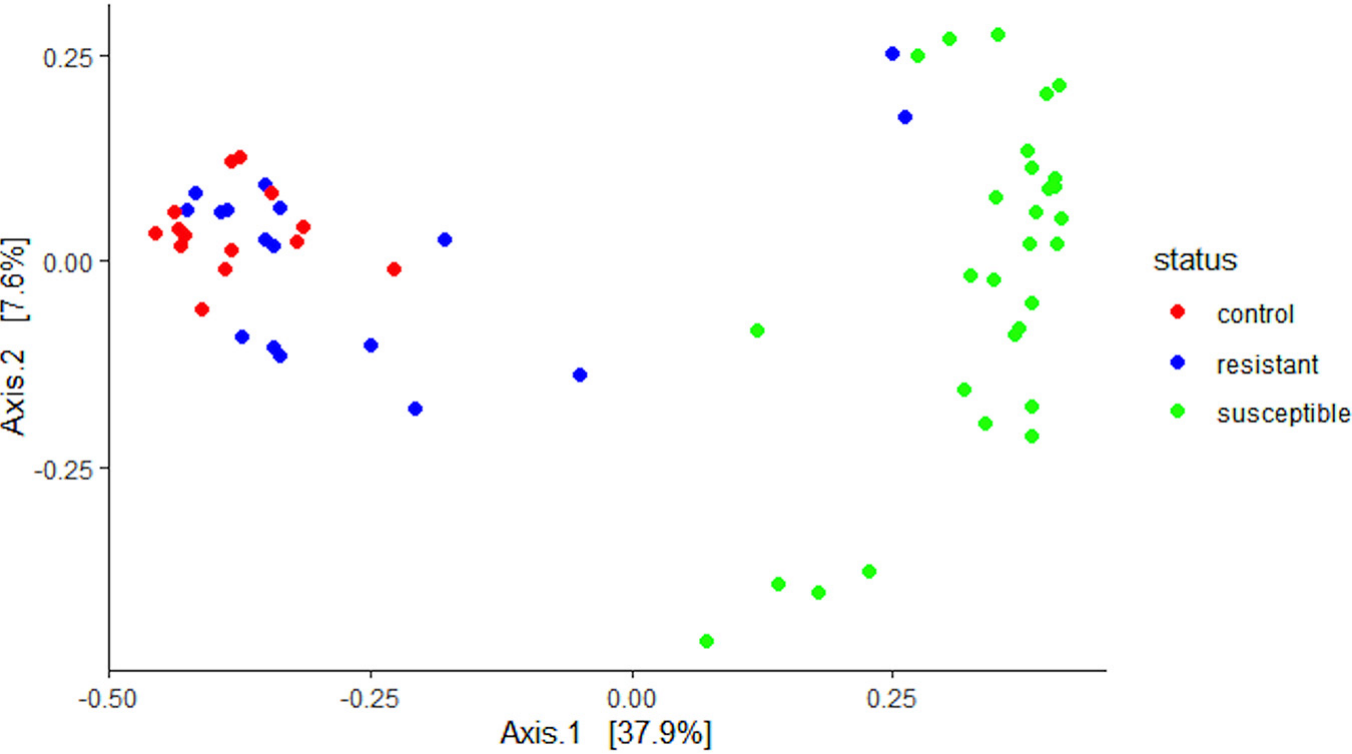
PCoA plot showing Bray-Curtis distance of microbiota between resistant, susceptible, and control F_1_ 2- to 3-day-old *An. coluzzii* adults. Each point represents the bacterial composition of a pool of three mosquitoes of the same resistance phenotype. There was a distinct separation between resistant/control and susceptible mosquitoes, which was shown to be a significant difference using a pairwise PERMANOVA (999 permutations) (pseudo-F = 19.44, *P* = 0.0015).

Comparison of the Bray-Curtis dissimilarity index using pairwise PERMANOVA with 999 permutations showed significant differences in bacterial composition between microbiota of 2- to 3-day-old deltamethrin-resistant and -susceptible *An. coluzzii* (pseudo-F = 19.44, *P* = 0.0015). Principal coordinate analysis (PCoA) visualizations showed the microbiota of susceptible mosquitoes clustered away from resistant and control mosquitoes (Fig. 1), indicating that the microbiota of susceptible mosquitoes were more similar to each other than to resistant and control mosquitoes.

Susceptible mosquitoes had significantly lower Shannon and Faith phylogenetic diversity indices (see Fig. S2) than resistant (Shannon: H = 13.91, q = 0.0003, Faith: H = 6.68, q = 0.01) and control (Shannon: H = 22.6 q = 0.000006, Faith: H = 16.6, q = 0.0001) mosquitoes of the same age, indicating that the susceptible group had reduced microbial diversity. There was no significant difference in alpha or beta diversity in deltamethrin-exposed and unexposed 5- to 6-day-old mosquitoes (Shannon: H = 5.12, q = 0.02, Faith: H = 0.27, q = 0.6, Bray-Curtis: pseudo-F = 1.61, q = 0.17), suggesting that insecticide exposure during the CDC bottle bioassays had minimal impact on microbial composition. There was no significant difference in alpha or beta diversity between mosquitoes exposed to 5 or 10 times the diagnostic dose of deltamethrin (Shannon: H = 0.05, q = 0.81, Faith: H = 0.68, q = 0.41, Bray-Curtis: pseudo-F = 0.96, q = 0.503).

### *Serratia* and *Asaia* dominated in older and younger susceptible *An. coluzzii*.

**FIG 2:**
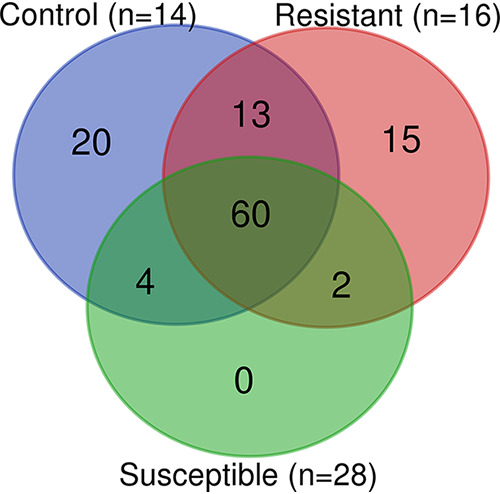
Venn diagram showing number of bacterial taxa unique to or shared between pools of 2- to 3-day-old resistant, susceptible, or control mosquitoes. Taxa were identified to genus level or lowest possible taxonomic rank. *n* = number of pools (each pool consists of three mosquitoes of the same age and phenotype).

There were significant differences in the microbiota of 2- to 3- and 5- to 6-day old mosquitoes (pseudo-F = 11.34, q = 0.001). Following taxonomic annotation of ASVs to the genus or lowest possible taxonomic level, 114 and 57 bacterial taxa were detected in 2- to 3-day-old and 5- to 6-day-old mosquitoes, respectively. The less diverse 5- to 6-day-old microbiota was predominantly comprised of ASVs assigned to the genera *Serratia* (75.5%) and *Asaia* (13.6%) (see Fig. S3). In 2- to 3-day-old mosquitoes, microbial composition varied by resistance phenotype. Control mosquitoes had the highest number of taxa present (*n* = 97), followed by resistant (*n* = 90) and susceptible (*n* = 66). A total of 20 taxa were unique to control mosquitoes, and 15 were unique to resistant mosquitoes. No taxa were unique to the susceptible group of mosquitoes, and 60 taxa were common to all groups (Fig. 2).

**FIG 3:**
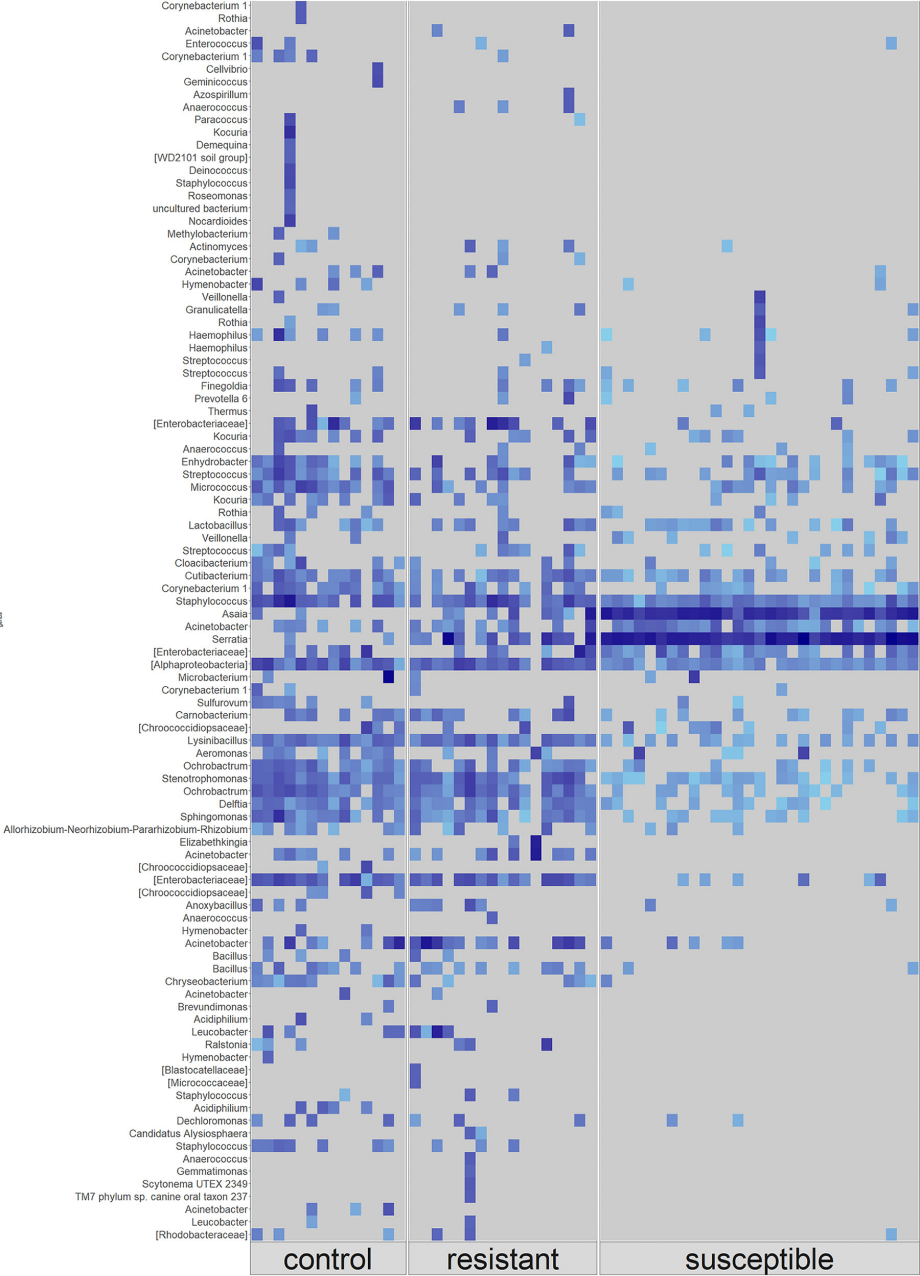
Raw frequency of ASVs from the microbiota of control (*n* = 14), resistant (*n* = 16), and susceptible (*n* = 28) F_1_ 2- to 3-day-old *An. coluzzii* adults. Each column represents a pool of three mosquitoes of the same phenotype. ASVs were annotated to genus level or lowest possible taxonomic level (in square brackets). Only taxonomically annotated ASVs with a frequency of >150 are shown. Light blue indicates a low frequency of ASVs present, while darker blue indicates a higher frequency. Gray indicates ASV was not present in that pool.

In control mosquitoes, an unclassified species within the *Enterobacteriaceae* family (15.24%), Acinetobacter (8.83%) and Staphylococcus (8.29%) were most abundant, while *Enterobacteriaceae* (15.12%), Acinetobacter (14.26%), and *Serratia* (11.8%) were the most abundant in resistant mosquitoes. In susceptible mosquitoes, *Serratia* (56.4%) and *Asaia* (30.92%) were the dominant genera, with Acinetobacter (1.96%), *Enterobacteriaceae* (1.57%), and Staphylococcus (1.4%) present at low abundance (Fig. 3). The remaining 61 taxa were present at an abundance of <1% of total ASVs present (see Fig. S3 and Table S4 in the supplemental material). Differential rankings confirmed that *Asaia* and *Serratia* were significantly associated with susceptibility and that *Stenotrophomonas*, *Ochrobactrum*, *Lysinibacillus*, and *Alphaproteobacteria* were significantly associated with phenotypic resistance.

**FIG 4:**
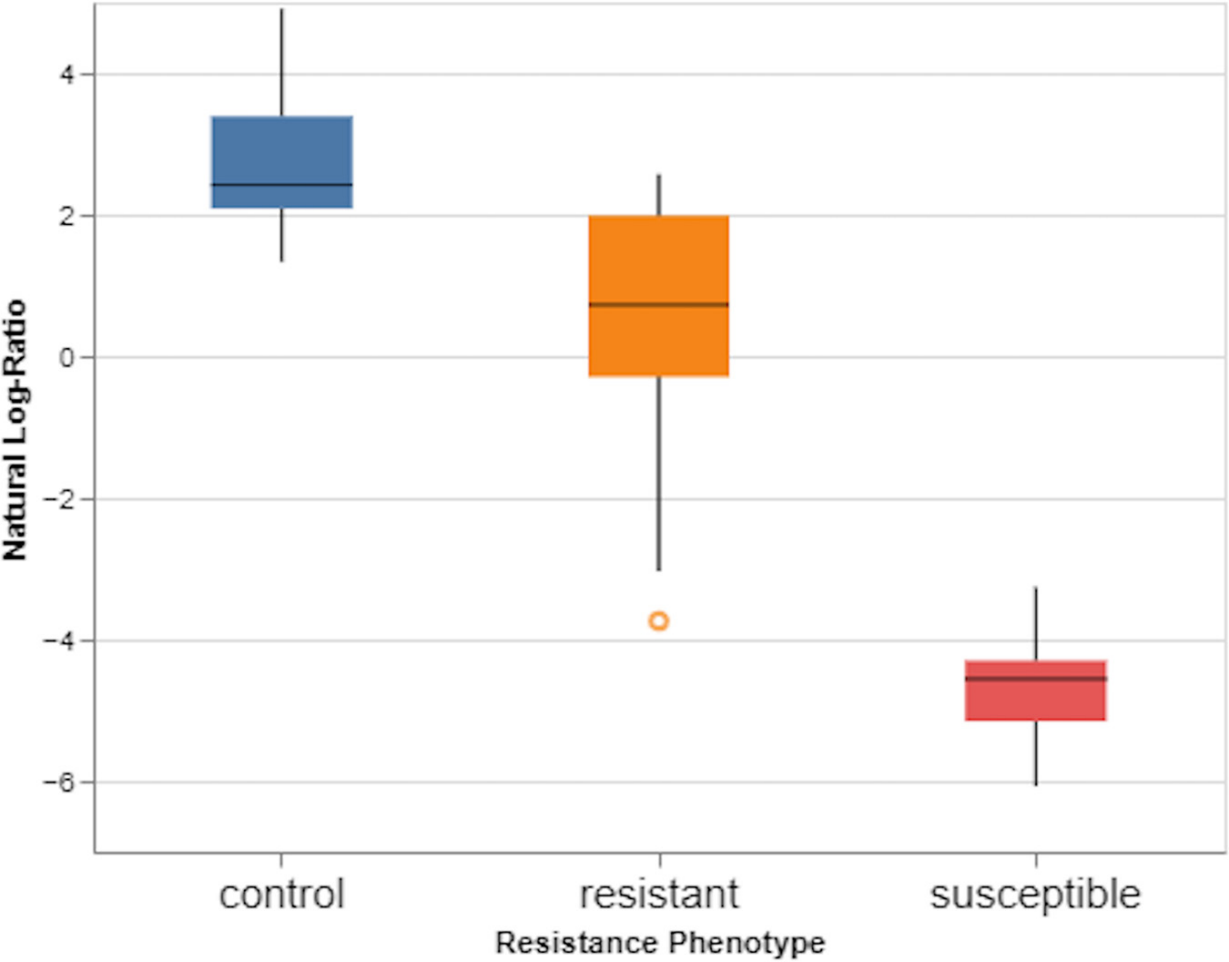
Log ratios of 10% highest ranked features to 10% lowest ranked features in control, resistant, and susceptible 2- to 3-day-old F_1_ *An. coluzzii*. Susceptible mosquitoes had a significantly lower ratio than control or resistant mosquitoes indicating that the lowest ranked features were overabundant in the susceptible group, while the highest ranked features were overabundant in either resistant or control mosquitoes.

Songbird was used to identify taxa which were differentially abundant in 2- to 3-day-old resistant, susceptible, or control mosquitoes. Evaluation of our Songbird model with resistance phenotype as the variable, against a baseline model with no variable resulted in a pseudo Q-squared value of 0.42, indicating that the model had not been overfit and that roughly 42% of variation in the model was predicted by resistance phenotype (see Fig. S4). There were significant differences in the log ratios of highest to lowest ranked taxa between resistant and susceptible microbiota (Fig. 4; see also Table S5), suggesting that the highest ranked taxa were significantly overabundant in resistant microbiota and that the lowest ranked taxa were significantly overabundant in susceptible microbiota.

**FIG 5:**
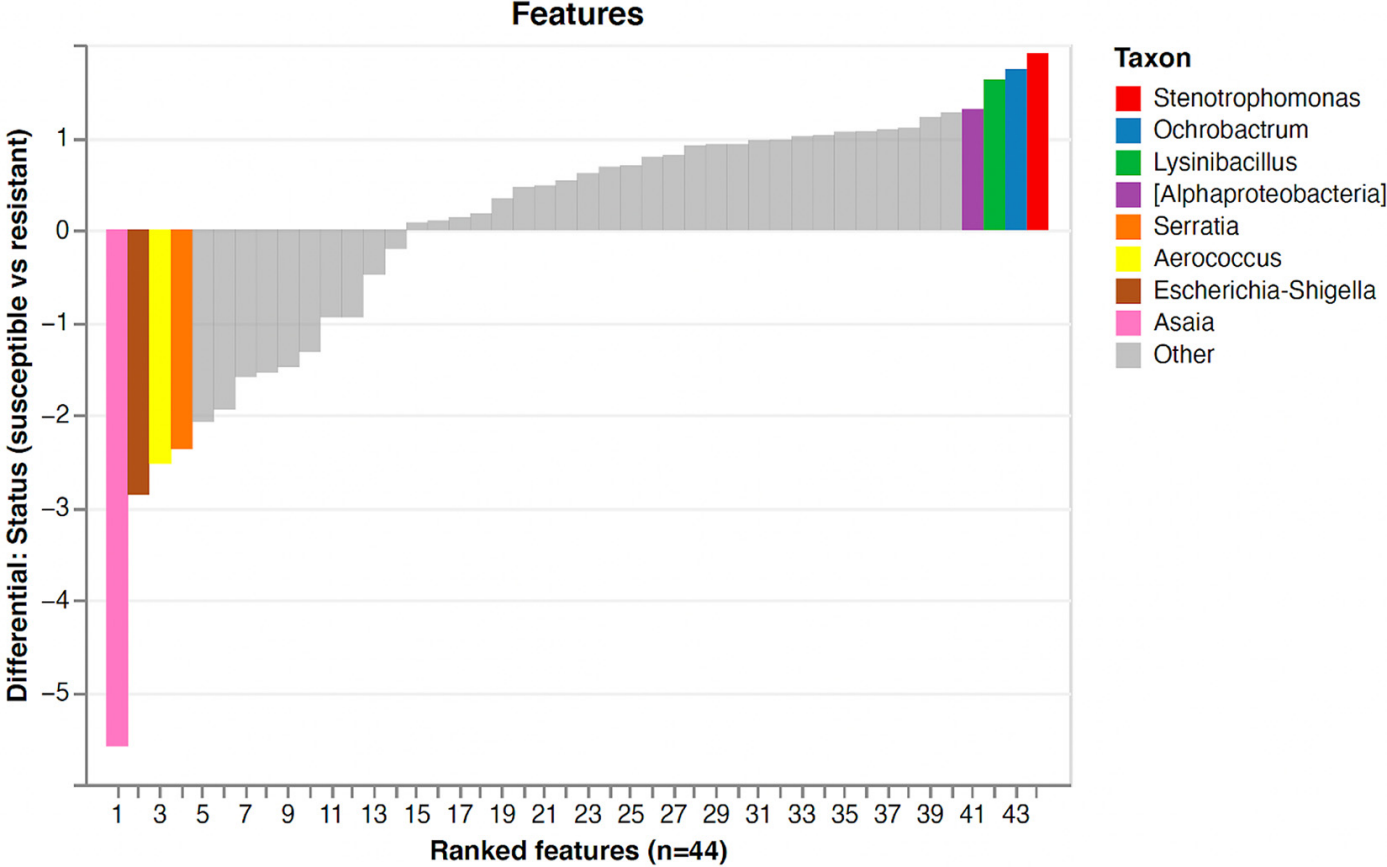
Sorted differential ranks of features associated with a resistant or a susceptible phenotype in 2- to 3-day-old *An. coluzzii*. The highest 10% and lowest 10% of ranked features are shown, colored by their corresponding assigned taxon. Taxa are shown to genus or to the lowest possible taxonomic level (square brackets).

*Stenotrophomonas*, *Ochrobactrum*, *Lysinibacillus*, and *Alphaproteobacteria* (highest ranked) were most strongly associated with insecticide resistance, while *Serratia*, *Aerococcus*, *E. shigella*, and *Asaia* (lowest ranked) were most strongly associated with insecticide susceptibility (Fig. 5). Comparing log ratios of control and susceptible pools indicated that *Rhodococcus*, *Sphingomonas*, Haemophilus, and *E. shigella* were most strongly associated with controls, while an uncultured *Chroocooccidiopsaceae*, *Serratia*, an unclassified member of *Enterobacteriacea*e, and *Asaia* were most strongly associated with susceptible mosquitoes (see Table S5).

These results were confirmed by the ANCOM method. *Serratia* (W = 208) and *Asaia* (W = 208) were significantly overabundant in susceptible mosquitoes relative to resistant and controls, while *Ochrobactrum* (W = 199), *Lysinibacillus* (W = 188), and *Enterobacteriaceae* (W = 201) were overabundant in resistant and control mosquitoes (see Fig. S5).

### Increased abundance of *Serratia* and *Asaia* species in susceptible individuals confirmed by qPCR.

Quantitative PCR (qPCR) assays confirmed that *Serratia* was significantly overabundant in 2- to 3-day-old susceptible mosquitoes compared to deltamethrin-resistant (5× *P* = 0.028; 10× *P* = 0.002) and control (*P* = 0.02) (average Δ*C_T_* values for susceptible, −8.4 [95% CI = −9.0 to –7.74]; 5×, –7.43 [–7.9 to –6.9]; 10×, –6.3 [–7.2 to –5.4]; and control, –7.2 [–7.9 to –6.6]) mosquitoes. *Asaia* was also significantly overabundant in 2- to 3-day-old susceptible mosquitoes compared to resistant (5×, *P* = <0.001; 10×, *P* < 0.001) and control (*P* < 0.001) (average Δ*C_T_* values for susceptible, –7.6 [95% CI = –8.5 to –6.8]; 5×, 0.8 [–1.6 to 3.2]; 10×, 5.1 [3.0 to 7.1]; and control, 4.5 [3.3 to 5.7]). Five- to six-day-old mosquitoes also had increased abundance of both bacterial species (average Δ*C_T_* value for 5- to 6-day-old 5×, S*erratia*, –8.2 [95% CI = –8.9 to –7.4] and *Asaia*, –1.3 [–5.6 to 2.9]; 5- to 6-day-old 10×, *Serratia*, –7.5 [–7.9 to –7.1] and *Asaia*, 5.4 [4.6 to 6.3]; 5- to 6-day-old control *Serratia*, –8.7 [–9.6 to –7.8] and *Asaia*, –0.68 [–3.0 to 1.7]).

## DISCUSSION

There is increasing evidence for an association between insecticide resistance phenotype (38, 41, 43) and *Anopheles* spp. microbiota. This study revealed distinct differences between the microbiota of deltamethrin-resistant and -susceptible *An. coluzzii*, with significant enrichment of insecticide degrading taxa in resistant individuals and an overabundance of *Serratia* and *Asaia* taxa in susceptible individuals. This population of field-caught *An. gambiae* s.l. from Agboville, Southeast Côte d’Ivoire, has previously been characterized as intensely resistant to pyrethroids, with average vector mortalities of 14.56% (95% CI = 8.92 to 22.88%), 61.62% (95% CI = 51.58 to 70.75%), and 73.79% (95% CI = 64.35 to 81.45%) to 1, 5, and 10 times the diagnostic doses of deltamethrin, respectively, and pyrethroid resistance associated with overexpression of CYP450 enzymes (*CYP6P4*, *CYP6Z1*, and *CYP6P3*) (44).

Our study demonstrated significant differences in alpha and beta diversity between deltamethrin-resistant and -susceptible *An. coluzzii.* Resistant mosquitoes harbored a wider variety of microbial taxa and had more microbial diversity both within and between themselves. Susceptible mosquitoes had fewer bacterial taxa and were far more homogenous, with *Serratia* and *Asaia* dominating in all samples. Previous studies have demonstrated differences between the microbiota of insecticide-resistant and -susceptible *An. stephensi* (38), *An. arabiensis* (42), and *An. gambiae* s.s. (43). This study is the first to detect an increase in alpha diversity in resistant mosquitoes, with other studies reporting no difference (39, 43) or a decrease (45). There are multiple potential reasons for the decreased microbial diversity previously identified, including processing of individuals rather than pooled mosquitoes, host species-specific differences, and variability among geographical collection sites (45). Furthermore, field-caught mosquitoes may be of unknown age and physiological status; prior environmental insecticide exposure might also be responsible for reducing overall diversity, as bacteria with the ability to metabolize insecticides have greater access to compounds for growth and reproduction and can outcompete other species (38). In our study, larvae were raised in distilled water, adults were age standardized, unable to mate, or blood-feed, and had no insecticide exposure prior to resistance assays. We therefore consider this inherited microbiota to be linked to the resistance status of the host, with mosquitoes characterized by a wider range of bacteria having an increased chance of harboring an insecticide degrading strain. Raising in sterile cups under identical rearing conditions reduces the environmental impact on the microbiome (45). In other organisms, particularly coprophagic animals such as rodents, individuals residing in the same cage have increasingly homogenous fecal microbiomes over time (46). It is plausible that the cage effect may have influenced the mosquito microbiota in this study, particularly during larval development where bacteria may be transferred between larvae via breeding water (21). The cage effect has not been widely studied in mosquitoes; however, future research should control for this in downstream analysis to minimize any environmental confounding.

Our study identified significant enrichment of several insecticide-degrading taxa in resistant compared to susceptible mosquitoes: *Ochrobactrum*, *Lysinibacillus*, and *Stenotrophomonas*. *Ochrobactrum* spp. have been isolated from contaminated soil and shown to degrade a variety of insecticides, including pyrethroids (47, 48) and organophosphates (49, 50). Similarly, *Lysinibacillus* spp. derived from soil and sewage can metabolize deltamethrin (51) and cyfluthrin (52), while *Stenotrophomonas* in the microflora of cockroaches living in pesticide-treated environments can degrade endosulfan *in vitro* (53). Elevated expression of xenobiotic degrading genes (51) and enzymes (38) may be contributing to the insecticide degrading properties of these bacteria, as well as the direct degradation of pesticides. Future work could attempt to isolate these bacteria from the mosquito microbiota and perform *in vitro* insecticide degradation assays, which would provide more evidence for bacterial degradation of pesticides, thus establishing a causal relationship between the microbiota and resistance phenotype.

While certain species of bacteria can confer insecticide resistance to the host, others may influence susceptibility. Indigenous gut bacteria have been implicated in the susceptibility of the gypsy moth, *L. dispar*, to the insecticidal toxin *Bacillus thuringiensis*. Treatment of larvae with antibiotics eliminated gut microbiota and subsequently reduced mortality to B. thuringiensis; susceptibility was restored upon oral administration of Enterobacter sp., a Gram-negative bacterium widely present in the *L. dispar* gut (54). In our study, *Serratia* and *Asaia* were found to be significantly overabundant in susceptible mosquitoes. While there are no prior reports of an association of these species with mosquito insecticide resistance phenotype, when the relative abundance of *Serratia* sp. in the gut of the diamondback moth was increased, susceptibility to chlorpyrifos significantly increased (55). Serratia marcescens plays a role in the susceptibility of field-caught *Aedes* to dengue virus infection by secreting *Sm*Enhancin, an enzyme which digests gut epithelia mucins, enabling the virus to penetrate the gut (35). The Bel protein, similar to *Sm*Enhancin and produced by the bacteria B. thuringiensis, has been shown to significantly increase the toxicity of Cry1Ac toxin in the cotton bollworm larvae, Helicoverpa armigera, by perforating the midgut peritrophic matrix and degrading the insect intestinal mucin, enabling the toxin to reach the target epithelial membrane (56). A similar mechanism may occur in these mosquitoes, whereby proteins produced by *Serratia* spp. increase the permeability of the internal organs to deltamethrin, enabling it to reach its target in the mosquito nervous system.

Insecticide resistance status has also been shown to influence P. falciparum development, with pyrethroid exposure adversely affecting L1014S-*kdr*-resistant *An. gambiae* s.s. in Uganda (57). Other studies have reported potentiation of *An. gambiae* vector competence in individuals carrying the *kdr* mutation (58). Elucidating the impact of mosquito host microbiota composition and molecular and metabolic resistance mechanisms on parasite infection dynamics is crucial for the design of novel transmission-blocking strategies. As well as being associated with resistance, *Asaia* and *Serratia* sp. have also both previously been implicated in the modulation of *Anopheles* vector competence. *Asaia* spp. have been shown to activate antimicrobial peptide expression in *An. stephensi* (59), while some strains of S. marcescens isolated from *An. sinensis* can inhibit *Plasmodium* development by altering the immunity-related effector genes *TEP1* and *FBN9* (27). *Serratia* spp. may also directly inhibit malaria parasite development by secretion of serralysin proteins and prodigiosin, which can have a pathogen-killing effect *in vitro* (60); the latter can also act as a larvicidal agent against *Ae. aegypti* and *An. stephensi* (61). By comparison, the presence of a dominant commensal *Enterobacteriaceae* has been positively correlated with *Plasmodium* infection (33).

No significant difference in the microbiota of deltamethrin-exposed and -unexposed mosquitoes was observed at 60 min or 72 h postexposure. One hour is likely insufficient time for the microbial composition to significantly shift in response to insecticide, given the relatively slow rate of bacterial growth, and the fact that any bacteria killed by insecticide exposure would still have been present during microbiota extraction. At 72 h, differences in the microbiota of exposed and unexposed mosquitoes, if present, should have been apparent. The lack of difference observed may in part be due to the low sample size of the 5-day-old 10×-resistant group and may also reflect the short insecticide contact time. Bioassays are a single exposure at a lethal dose in a sterile environment used to determine resistance phenotype (62). In the wild, multiple insecticide exposures are likely to happen at sublethal doses at both the larval stage, as habitats are contaminated with agricultural pesticides, and the adult stage, as there is frequent interaction with treated surfaces or materials indoors (63). Bioassays may therefore not induce the same shifts in microbiota as insecticide exposure in the wild. Furthermore, as mosquitoes acquire most of their microbiota from the aquatic environment at the larval stage (21) and may obtain insecticide metabolizing bacteria at this stage, studying the effects of deltamethrin exposure on larvae, or adults which were exposed as larvae, may be more informative.

Our results demonstrated a significant relative reduction in alpha diversity in resistant and control 5- to 6-day-old mosquitoes compared to the 2- to 3-day-old group, as expected based on prior reports that microbial diversity declines with age (64). Older mosquitoes also had lower relative abundances of *Ochrobactrum*, *Lysinibacillus*, and *Stenotrophomonas*, the insecticide-degrading species shown to be significantly enriched in resistant 2- to 3-day-old individuals. *Serratia* and *Asaia*, the species associated with susceptibility, were present in increased abundance in the older age group. It has been widely reported that insecticide resistance declines with age (65–70). The shift in microbiota may also be a contributing factor, and further research is warranted to determine the association between the microbiota, resistance phenotype, and age.

In our study, qPCR detection of *Serratia* and *Asaia* was consistent with the 16S rRNA sequencing data, with susceptible mosquitoes having significantly lower Δ*C_T_* values than resistant or control individuals. Population-level field screening using qPCR as a less-expensive, faster, and more-feasible option than amplicon sequencing should be considered for integration in wider insecticide resistance monitoring, if reliable and reproducible bacterial markers associated with phenotype can be identified.

### Conclusions.

Insecticide susceptibility is influenced by a range of diverse factors, including host genetics, detoxification systems, and behavior, as well as the mosquito microbiota. We report significant differences in the microbiota of deltamethrin-resistant *An. coluzzii* and have identified several bacterial species that were associated with either resistance or susceptibility to the host and therefore may represent important markers of resistance phenotype. The role of bacteria in determining resistance phenotype is highly complex and specific to the host and bacterial species and insecticide and likely involves multiple, parallel mechanisms, including direct degradation of insecticide, an altered host immune system, and changes to the midgut. In addition, these interactions may have important implications for host species fitness, vector competence, and pathogen development and transmission. Further investigation into the mechanisms of microbiota-mediated susceptibility is necessary since this may provide opportunities for preventing or reducing insecticide resistance, which is crucial to maintain gains in malaria vector control.

## MATERIALS AND METHODS

### Mosquito collections and mass rearing.

This study was conducted in Agboville (GPS: 5°55′21″N 4°13′13″W), Agnéby-Tiassa region, southeast Côte d’Ivoire. The location was chosen because of its high mosquito densities, malaria prevalence (26% in children <5 years in 2016 [71]), and intense deltamethrin resistance (44). The main industry is agriculture, with livestock such as cows, goats, and chickens living close to households and cultivation of crops, including bananas, cocoa, and rice (72).

Sampling was conducted between 5 July and 26 July 2019, coinciding with the long rainy season (May-November) and peak malaria transmission. Adult mosquitoes were collected using HLCs, inside and outside households from 18:00 h to 06:00 h. Fieldworkers used individual hemolysis tubes to collect host-seeking mosquitoes, which were transported each morning to the Centre Suisse de Recherche Scientifique en Côte d’Ivoire (CSRS) in Abidjan. Blood-fed mosquitoes, morphologically identified as female *An. gambiae* s.l. (73), were transferred to cages with 10% sugar solution and left for 2 to 3 days to become fully gravid.

A total of 580 fully gravid females were used for forced oviposition. Oviposition was achieved by placing a single gravid mosquito into a 1.5-ml Eppendorf tube, half filled with damp cotton wool, with small holes in the tube cap for ventilation (74). Mosquitoes were held under standard insectary conditions (25°C, 70% humidity, and a 12-h light-dark cycle) until eggs were laid or adult death. Eggs were removed daily and placed into sterile paper cups containing distilled water and NISHIKOI (Nishikoi, United Kingdom) (75) staple fish food pellets. Emergent larvae were reared in 50-cm washing-up bowls in distilled water under the same insectary conditions. Pupae were removed daily and separated by sex with the aid of a stereomicroscope. Female pupae were put in a clean plastic cup with distilled water and placed in a cage for eclosion, while male pupae were discarded. Adults were housed in cages in an incubator (26.6°C, 70% humidity) with a 12-h light-dark cycle and given unlimited access to 10% glucose solution. The cages were checked to ensure that only virgin females were used in bioassays, since mating can potentially introduce changes to the microbiome (20). Care was also taken to ensure that no mosquito obtained a blood meal during handling, since this can significantly decrease bacterial diversity in the gut (64).

### Determining deltamethrin resistance status of adult F_1_ progeny of field-caught *An. gambiae* s.l.

Deltamethrin resistance was characterized using Centers for Disease Control (CDC) bottle bioassays (62), with some modifications. Two- to three-day-old virgin F_1_ females were exposed to 1, 5, or 10 times the diagnostic dose of deltamethrin (12.5 μg/bottle) for 30 min. Stock solutions of deltamethrin were prepared using 100% ethanol as the solvent. Per bioassay, multiple 250-ml Wheaton bottles and their lids were coated with 1 ml of stock solution and left to dry in a dark storage area to avoid exposure to UV light. A control bottle, treated with 1 ml of ethanol, was assayed in parallel. Prior to bioassay testing, approximately 20 to 25 mosquitoes were aspirated into holding cups. After 1 to 2 h of acclimatization, they were introduced into each test or control bottle.

Knockdown was scored at 0, 15, and 30 min. A subset of mosquitoes that were alive at 60 min was held for 72 h, with mortality recorded every 24 h. These mosquitoes were housed in paper cups in the insectary, with unlimited access to sterile 10% glucose made with distilled water. Mosquitoes were counted as dead if they were unable to stand as per WHO criteria (62).

At the end of the bioassay and subsequent holding time, mosquitoes were classified as susceptible if they were knocked down following exposure to 1× deltamethrin; resistant if they survived 60 min or 72 h postexposure to 1×, 5×, or 10× deltamethrin; or controls if they were in the ethanol-coated bottle. Specimens were separated into their respective phenotype and concentration/time group and stored at −70°C.

### DNA extraction.

DNA was extracted from 380 mosquitoes that had been categorized as resistant, susceptible, or unexposed to deltamethrin. Individuals were homogenized in a Qiagen TissueLyser II with sterilized 5-mm stainless steel beads for 5 min at 30 Hz and incubated overnight at 56°C. DNA was extracted using a Qiagen DNeasy 96 blood and tissue kit (Qiagen, UK) according to the manufacturer’s protocol (76) with DNA eluted in 45 μl of buffer AE. Extracted DNA was stored at −70°C.

Four blank extraction controls were processed alongside mosquitoes: three blanks containing RNase-free water as the extraction template and one blank containing the 70% ethanol used for reagent dilution and sterilization of instruments. All steps were performed under sterile conditions, with tweezers and other instruments being rinsed with 70% ethanol in between handling each mosquito, to avoid microbial or DNA contamination.

### PCR for mosquito species identification.

Individual mosquitoes were identified to species level according to Santolamazza et al. (77). PCRs contained 2 μl of 10 μM forward primer (5′-TCGCCTTAGACCTTGCGTTA-3′), 2 μl of 10 μM reverse primer (5′-CGCTTCAAGAATTCGAGATAC-3′), 1 μl of extracted DNA, and 10 μl of Hot Start Taq 2X Master Mix (New England Biolabs, UK) for a final reaction volume of 20 μl. Prepared reactions were run on a Bio-Rad T100 thermal cycler with the following conditions: 10 min denaturation time at 94°C, followed by 35 amplification cycles of 94°C for 30 s, 54°C for 30 s, and 72°C for 60 s, followed by a final extension at 72°C for 10 min. PCR products were visualized on 2% E-gel agarose gels in an Invitrogen E-gel iBase real-time transilluminator. A Quick-Load 100-bp DNA ladder (New England Biolabs) was used to determine band size. Amplified PCR products of 479 or 249 bp were indicative of *An. coluzzii* or *An. gambiae* s.s., respectively. As the dominant species, only *An. coluzzii* individuals of the same age and resistance phenotype were selected and pooled for 16S rRNA sequencing.

### 16S rRNA gene amplicon sequencing.

DNA concentration from each mosquito was measured using an Invitrogen Qubit 4 fluorometer (Thermo Fisher Scientific, USA). Pools were prepared by combining equal concentrations of DNA from three mosquitoes of the same phenotype/deltamethrin concentration/time group to give 100 ng in a final volume of 20 μl (see Table S1 in the supplemental material). Two negative controls, one comprised of a pool of the three RNase-free water blanks mentioned above and the other a 70% ethanol blank, were processed in parallel.

The microbial composition of the microbiome was determined by amplification of the V3-V4 region of the *16S rRNA* gene, using the primers 5′-CCTACGGGNGGCWGCAG-3′ and 5′-GGACTACHVGGGTATCTAATCC-3′. PCRs were prepared in a 25 μl reaction volume, comprising 12.5 μl of KAPA HiFi Hot Start ReadyMix PCR kit (78) (Roche, Switzerland), 0.5 μl of forward and reverse primers (10 μM), and 12.5 ng of DNA. The following PCR cycling was used: 95°C for 3 min and 35 cycles of 95°C for 30 s, 55°C for 30 s, and 72°C for 30 s, followed by a final extension at 72°C for 5 min. The resulting amplified PCR products were purified with AMPure XP beads (Beckman Coulter, UK) at 1× sample volume.

Next, an index PCR was performed using 5 μl of purified PCR products, 5 μl of Nextera XT Index 1 Primers (N7XX), 5 μl of Nextera XT Index 2 Primers (S5XX) (both from the Nextera XT Index kit; Illumina, USA), 10 μl PCR grade water and 25 μl of KAPA HiFi Hot Start Ready Mix (Roche, Switzerland). The following PCR cycling was used: 95°C for 3 min and 12 cycles of 95°C for 30 s, 55°C for 30 s, and 72°C for 30 s, followed by a final extension at 72°C for 5 min. The final library was purified with AMPure XP beads, at a 1.12× sample volume, before quantification.

Sequencing was performed on an Illumina MiSeq platform. Libraries were sequenced as 250-bp paired-end reads. Sequences were demultiplexed and filtered for read quality using Bcl2Fastq conversion software (Illumina, Inc.). In total, 1,156,076 sequences were generated in the FASTQPhred33 format44.

### Data cleaning and filtering.

Sequencing data were imported into the “Quantitative Insights Into Microbial Ecology” pipeline, version 2020.8 (Qiime2) (79), and primary analysis was performed on the reverse reads, as the quality of the forward reads was not sufficient for merging (see Fig. S1). Sequencing primers and adapters were removed using the “cutadapt” plugin (80) with an error rate of 10%. The divisive amplicon denoising algorithm (DADA2) plugin (81) was used to “denoise” sequencing reads, removing phiX reads and chimeric sequences, to produce high-resolution ASVs (82). DADA2 was run using the denoise-single command, with samples truncated at 206 nucleotides (trunc-len 206), to remove bases with a low-quality score. All other parameters were set to the default. The resulting feature table (83) and sequences were filtered to remove ASVs present in the two blank samples and those with a frequency of below 100 to reduce biases in comparison of diversity indices across groups and especially in differential abundance tests.

### Taxonomic annotation.

Taxonomic annotation of ASVs was performed using the -feature-classifier plugin (84), with a Naive-Bayes classifier (85) pretrained on the *16S* SILVA reference (99% identity) database version 132. The -extract-reads command was used to trim the reference sequences to span the V3-V4 region (425 bp) of the *16S rRNA* gene. Any features not classified to phylum level were also removed, these included hosts’ mitochondrial *16S rRNA* genes. The resulting ASV table was exported into R (version 3.6.3) for analysis with the phyloseq package.

### Bacterial diversity analysis.

A rooted and unrooted phylogenetic tree was generated using the qiime phylogeny plugin (86–88) and were used to compute alpha and beta diversity metrics using the qiime2-diversity (89) plugin. For alpha diversity metrics, samples were rarefied (90) at a depth of 2,359, where alpha rarefaction curves plateaued, indicating that there was adequate sampling of the microbiota during sequencing. Beta diversity metrics were computed for both rarefied and nonrarefied data, with no significant differences between methods (see Table S2); nonrarefied data are presented here. Mosquitoes 2 to 3 days old and 5 to 6 days old were analyzed separately, since age was shown to significantly impact the bacterial composition of the microbiota. Mosquitoes resistant to 5 and 10 times the diagnostic dose of deltamethrin were analyzed together since the insecticide concentration was not shown to significantly impact the bacterial composition of resistant mosquitoes.

Two methods of alpha diversity were selected: the Shannon diversity Index, which considers the abundance and evenness of ASVs present, and the Faith phylogenetic diversity, a measure of community richness which incorporates phylogenetic relationships between species. Pairwise Kruskal-Wallis comparisons of these alpha diversity indices between groups of insecticide resistance phenotypes were performed, with a Benjamini-Hochberg false discovery rate (FDR) correction for multiple comparisons (91). Significance was set to the FDR adjusted *P* value, i.e., *q* < 0.05.

The Bray-Curtis dissimilarity index (92, 93), which measures differences in relative species composition between samples, was chosen as the beta diversity metric. Comparisons of this index between insecticide resistance phenotype groups were conducted using pairwise PERMANOVA tests with 999 permutations (94). Results were visualized using PCoA generated using the phyloseq (95) package. Significance was set to a *P* value of <0.05.

### Determination of association between microbiota composition and insecticide resistance phenotype, and identification of differentially abundant microbial taxa.

Comparison of alpha and beta diversity indices indicated that both insecticide resistance phenotype and mosquito age affected the bacterial composition of *An. coluzzii* in this study. Following taxonomic annotation of ASVs, multinomial regression and differential abundance analysis was performed using Songbird (96) to determine the microbial taxa that were associated with and differentially abundant across insecticide resistance phenotype for mosquitoes separated by age group. Songbird is a compositionally aware differential abundance method which ranks features based on their log fold change with respect to covariates of interest (96) The following Songbird parameters were used: epochs, 10000; number of random test examples, 15; and differential prior, 0.5. The fit of the model was tested against the null hypothesis (-p-formula “1”). Differential log ratios of features were computed in Qurro (97). We present the highest and lowest 10% ranked features associated with resistance phenotype. The analysis of composition of microbiome method (ANCOM) was used to complement Songbird analysis, and this was computed using the composition plugin (98) with all parameters set to the default. Significance was determined using the automatic cut off for the test statistic, W (98).

### Quantitative PCR validation of sequencing data.

The abundance of *Serratia* spp. and *Asaia* spp. was assessed using qPCR, relative to the nuclear single-copy *An. gambiae* s.l. ribosomal protein S7 housekeeping gene (*RPS7*). *Serratia* reactions contained 1 μl of 10 μM forward primer (5′-CCGCGAAGGCAAAGTGCACGAACA-3′), 1 μl of 10 μM reverse primer (5′-CTTGGCCAGAAGCGCACCATAG-3′) (99), 2 μl of pooled DNA, and 5 μl LightCycler 480 SYBR green Master Mix (Roche, UK) for a final reaction volume of 10 μl. Prepared reactions were run on an Agilent Technologies Stratagene Mx3005P qPCR system which performed 40 cycles of 95°C for 15 s and 60°C for 1 min, followed by a dissociation curve. *Asaia* reactions contained 1 μl of 10 μM forward primer (5′-GCGCGTAGGCGGTTTACAC-3′), 1 μl of 10 μM reverse primer (5′-AGCGTCAGTAATGAGCCAGGTT-3′) (100), 2 μl of pooled DNA, and 5 μl of LightCycler 480 SYBR green Master Mix (Roche, UK) for a final reaction volume of 10 μl. Prepared reactions were run on an Agilent Technologies Stratagene Mx3005P qPCR system with the following conditions: 95°C for 15 min and 40 cycles of 95°C for 10 s, 60°C for 10 s, and 72°C for 10 s, followed by a dissociation curve. *RSP7* reactions contained 1 μl of 10 μM forward primer (5′-TCCTGGAGCTGGAGATGAAC-3′), 1 μl of 10 μM reverse primer (5′-GACGGGTCTGTACCTTCTGG-3′) (101), 2 μl of pooled DNA, and 5 μl of LightCycler 480 SYBR green Master Mix (Roche, UK) for a final reaction volume of 10 μl. Prepared reactions were run on an Agilent Technologies Stratagene Mx3005P qPCR system with the following conditions: 40 cycles of 95°C for 10 s, 65°C for 60 s, and 97°C for 1 s, followed by a dissociation curve. All samples were run in technical triplicate. Relative bacterial abundance was normalized relative to the endogenous control gene (*RPS7*). qPCR results were analyzed using the MxPro software (Agilent Technologies).

### Ethics approval and consent to participate.

The study protocol was reviewed and approved by the Comite National d’Ethique des Sciences de la Vie et de la Sante (catalog no. 069-19/MSHP/CNESVS-kp) and the institutional review board (IRB) of the London School of Hygiene and Tropical Medicine (no. 16860); all study procedures were performed in accordance with relevant guidelines and regulations. Prior to study initiation, community consent was sought from village leaders and written, informed consent was obtained from the heads of all households selected for participation and from all fieldworkers who performed HLCs. Fieldworkers participating in HLCs were provided with doxycycline malaria prophylaxis.

### Data availability.

Sequence data generated by this study is available at Sequence Read Archive (SRA) BioProject PRJNA702915 (accession numbers: SRR13743435 to SRR13743530). All other relevant data are available from the corresponding author upon reasonable request. Codes can be accessed at the public repository Zenodo (http://zenodo.org) under https://doi.org/10.5281/zenodo.5102954.
