# Supplementary material for: Overabundance of Asaia and Serratia Bacteria Is Associated with Deltamethrin Insecticide Susceptibility in Anopheles coluzzii from Agboville, Côte d’Ivoire
Source: Microbiol Spectr. 2021 Oct 20;9(2):e00157-21. doi: 10.1128/Spectrum.00157-21 (PMC8528120; doi:10.1128/Spectrum.00157-21)
Supplement: SUPPLEMENTAL FILE 1 — Supplemental material. Download SPECTRUM00157-21_Supp_1_seq7.pdf, PDF file, 0.6 MB [file spectrum00157-21_supp_1_seq7.pdf]

Description of Supplementary Files

File name: **Table S1. Numbers of mosquitoes selected for DNA extraction and 16S rRNA sequencing.** Mosquitoes were allocated to a concentration/time group based on the concentration of deltamethrin to which they were exposed, the period of follow up post exposure and whether they survived exposure. A selection from each group were pooled in groups of three and sequenced.

File name: **Table S2. Summary statistics of raw sequencing outputs and resulting data following denoising with Dada2 and filtering**.

File name: **Table S3. Relative abundances of bacterial species present in mosquitoes, grouped by age and resistance status.** Taxa are annotated to genus or lowest possible taxonomic level (square brackets).

File name: **Table S4. Beta diversity outputs, showing no significant difference between rarefied and non-rarefied data.** Samples which fail to meet the rarefaction depth are excluded from analysis, therefore the sample sizes of rarefied data are reduced.

File name: **Table S5. Differential rankings for features and taxa present in 2-3 day old *An. coluzzii,* as computed by Songbird.**

File name: **Figure S1. Quality plots for forward and reverse untrimmed reads, generated in Qiime2.** Low quality forward reads resulted in sequence loss due to failure of strands to merge during denoising, therefore only the reverse reads were used for downstream analysis.

File name: **Figure S2. Box plots showing Shannon index of diversity and Faith's Phylogenetic Diversity in 2-3 day old *An. coluzzii.*** Upper and lower limits of boxes represent first and third quintiles of the median (dark horizontal line).

File name: **Figure S3. Stacked bar plot showing relative abundance of taxa present in 2 day old and 5 day old *An. coluzzii* grouped by resistance phenotype.** Taxa are annotated to genus or lowest possible taxonomic level. Taxa present at less than 1% abundance are grouped together and coloured in grey.

File name: **Figure S4. Songbird model.** Blue line is trained on the dataset and is a predictor of model accuracy in contrast to the null hypothesis (orange). Illustrates the accuracy of model prediction and proportion of variance in microbial composition explained by resistance phenotype.

File name: **Figure S5. Volcano plot of differentially abundant bacterial taxa in 2-3 day old *An. coluzzii***, as computed by analysis of composition of microbiomes (ANCOM).

Table S1: Numbers of mosquitoes selected for DNA extraction and 16S rRNA sequencing. Mosquitoes were allocated to a concentration/time group based on the concentration of deltamethrin to which they were exposed, the period of follow up post exposure and whether they survived exposure. A selection from each group were pooled in groups of three and sequenced.

Resistance phenotype	Description	Concentration /time group	Age at death	Number of individuals	Number selected for sequencing (#pools)
Susceptible	Knocked-down at 60 minutes post exposure to the diagnostic dose (1x) deltamethrin.	Susceptible	2-3 days	97	87 (29)
Resistant	Survivors at 60 minutes post exposure to 5x deltamethrin	5xRes60	2-3 days	83	30 (10)
	Survivors at 60 minutes post exposure to 10x deltamethrin	10xRes60	2-3 days	89	21(7)
	Survivors at 72 hours post exposure to 5x deltamethrin	5xRes72	5-6 days	24	24(8)
	Survivors at 72 hours post exposure to 10x deltamethrin	10xRes72	5-6 days	33	12 (4)
	Survivors at 72 hours post exposure to diagnostic dose (1x) deltamethrin	1xRes72	5-6 days	55	36 (12)
Controls	Mosquitoes from control bottle which were alive 60 minutes after the initiation of bioassay	ControlA60	2-3 days	57	42 (14)
	Mosquitoes from control bottle which were alive 72 hours after the initiation of bioassay	ControlA72	5-6 days	69	30 (10)
Blanks	70% Ethanol which underwent same DNA extraction and 16S rRNA sequencing as samples	Ethanol blank	N/A	1	1 (1)
	Sterile, RNAse free water which underwent same DNA extraction and 16S rRNA sequencing as samples	Water blank		3	3 (1)

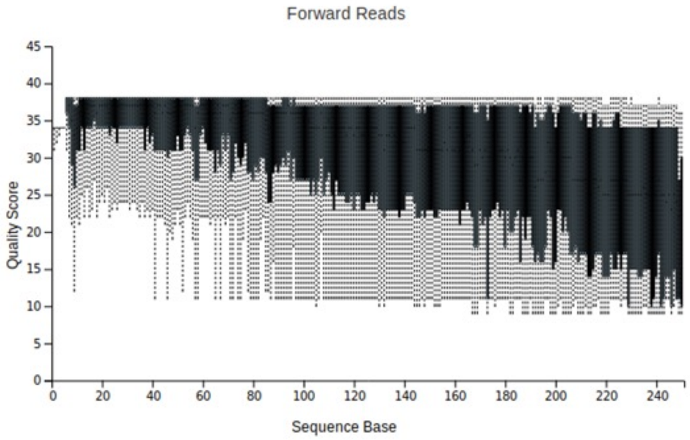

Forward Reads
Quality Score
Sequence Base
0
5
10
15
20
25
30
35
40
45
0
20
40
60
80
100
120
140
160
180
200
220
240

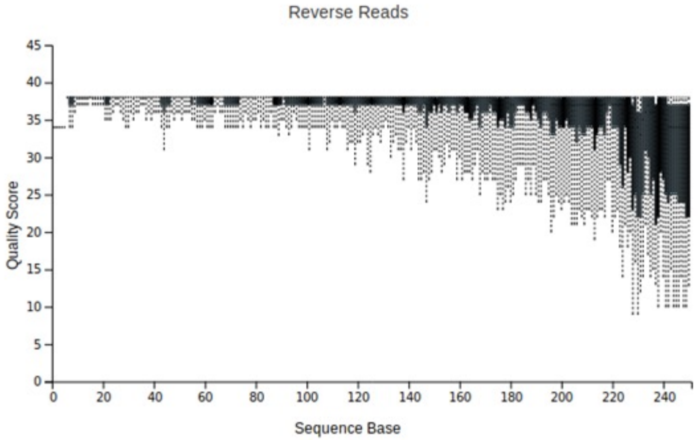

Reverse Reads
Quality Score
Sequence Base
0
5
10
15
20
25
30
35
40
45
0
20
40
60
80
100
120
140
160
180
200
220
240

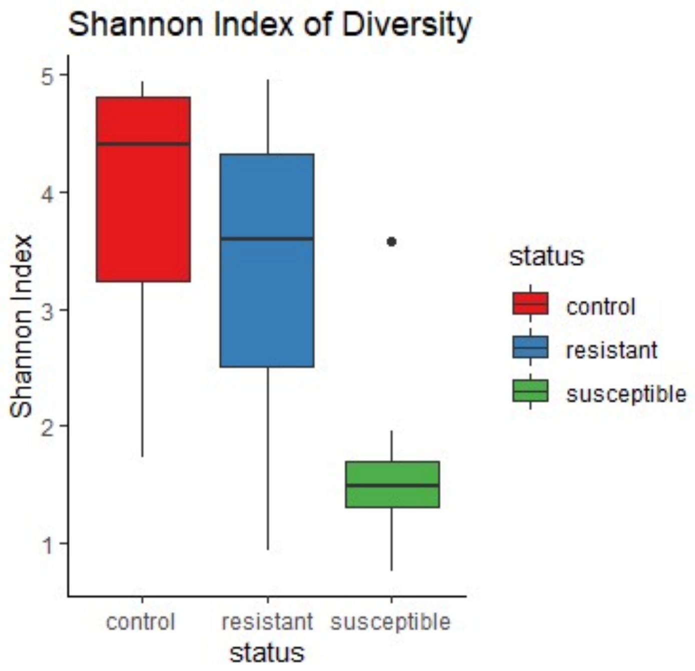
Shannon Index of Diversity
Shannon Index
5
4
3
2
1
control
resistant
susceptible
status
status
control
resistant
susceptible

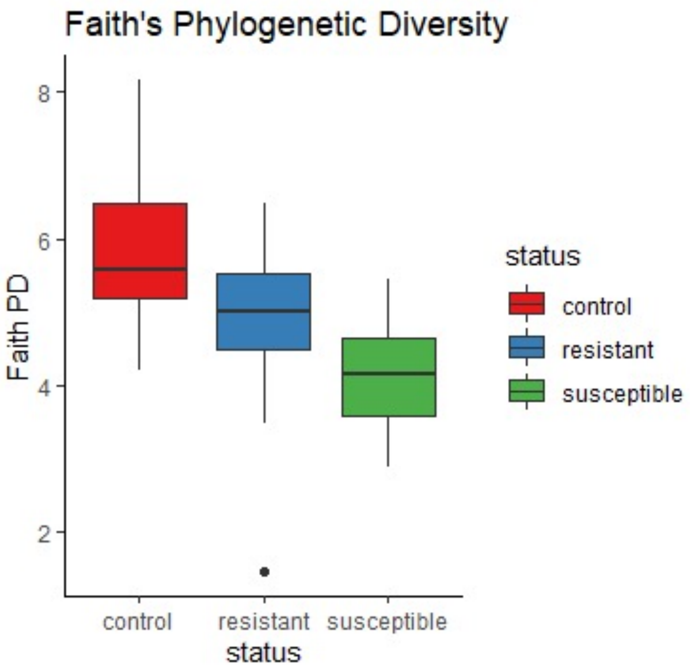
Faith's Phylogenetic Diversity
Faith PD
8
6
4
2
control
resistant
susceptible
status
status
control
resistant
susceptible

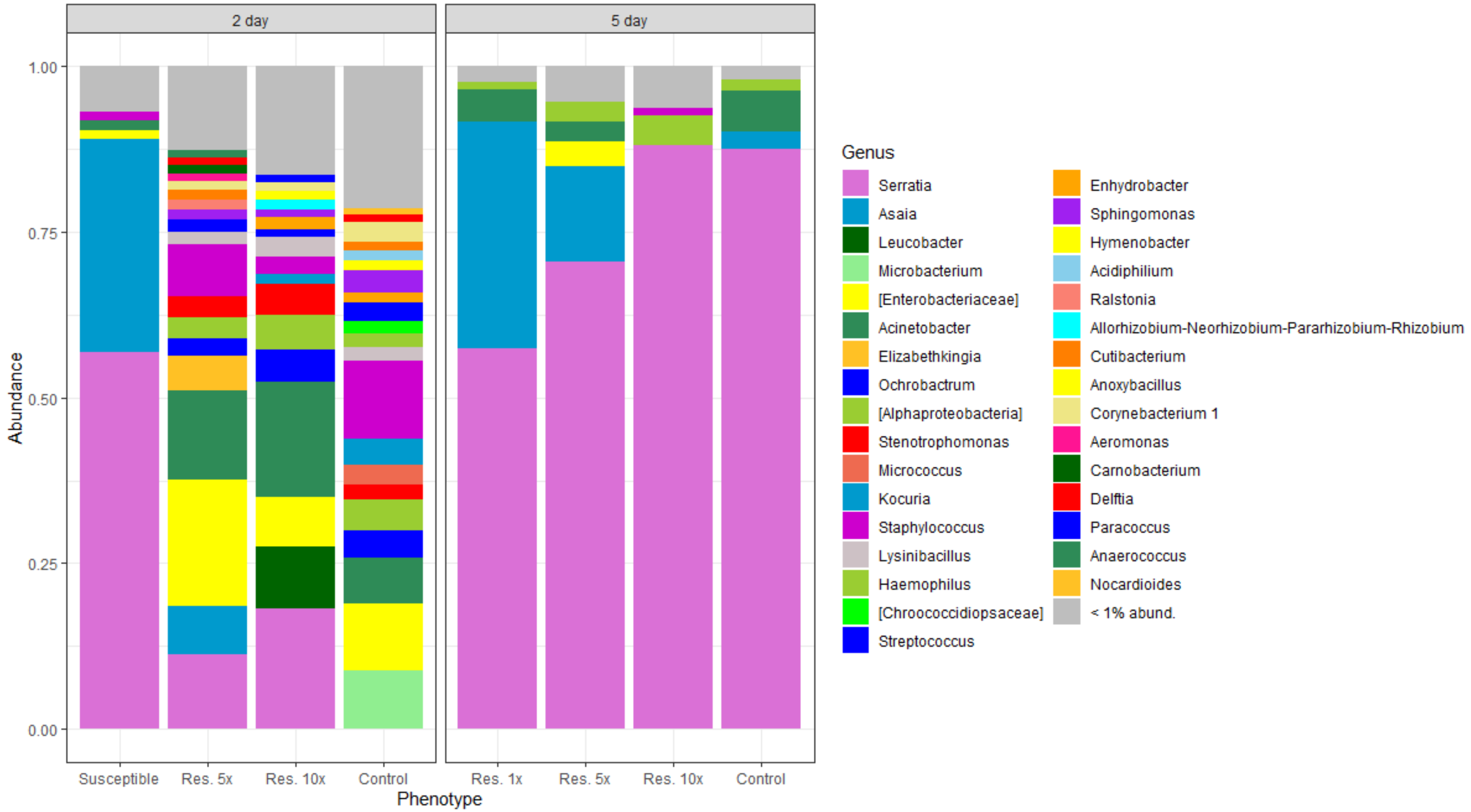
2 day
5 day
1.00
0.75
0.50
0.25
0.00
Abundance
Susceptible
Res. 5x
Res. 10x
Control
Res. 1x
Res. 5x
Res. 10x
Control
Phenotype
Genus
Serratia
Asaia
Leucobacter
Microbacterium
[Enterobacteriaceae]
Acinetobacter
Elizabethkingia
Ochrobactrum
[Alphaproteobacteria]
Stenotrophomonas
Micrococcus
Kocuria
Staphylococcus
Lysinibacillus
Haemophilus
[Chroococcidiopsaceae]
Streptococcus
Enhydrobacter
Sphingomonas
Hymenobacter
Acidiphilium
Ralstonia
Allorhizobium-Neorhizobium-Pararhizobium-Rhizobium
Cutibacterium
Anoxybacillus
Corynebacterium 1
Aeromonas
Carnobacterium
Delftia
Paracoccus
Anaerococcus
Nocardioides
< 1% abund.

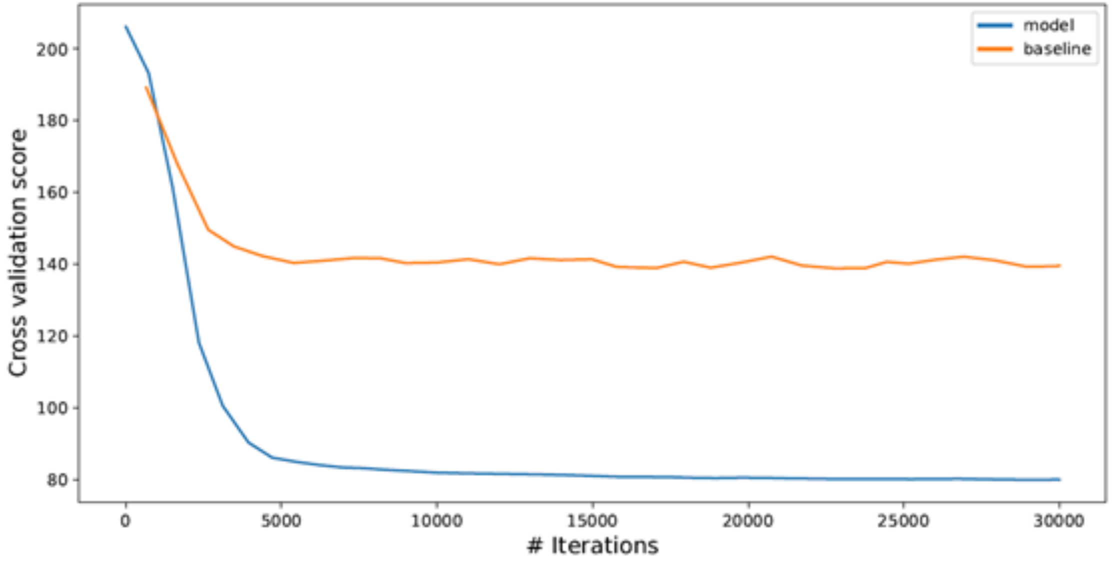
model
baseline
200
180
160
140
120
100
80
Cross validation score
0
5000
10000
15000
20000
25000
30000
Iterations

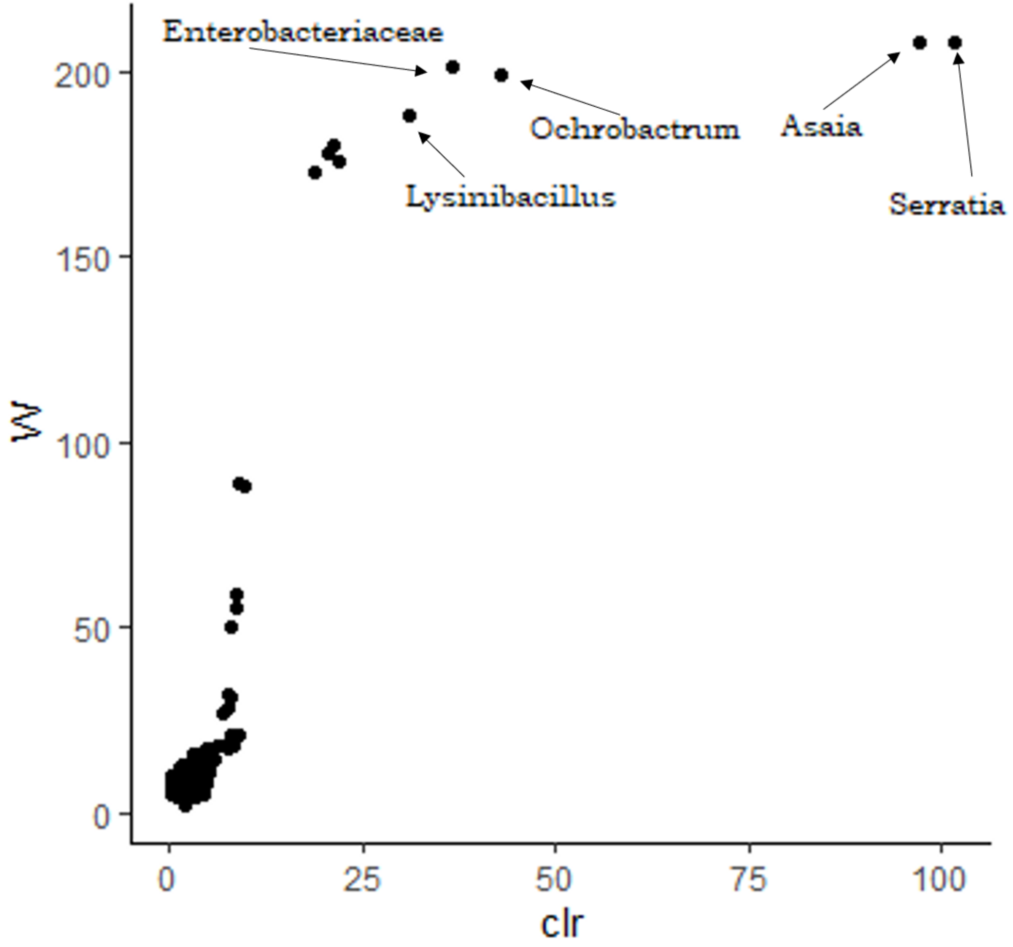

Enterobacteriaceae
Ochrobactrum
Asaia
Lysinibacillus
Serratia
200
150
100
50
0
W
0
25
50
75
100
clr
